# Subjective Sleep Quality in Amnestic Mild Cognitive Impairment Elderly and Its Possible Relationship With Plasma Amyloid-β

**DOI:** 10.3389/fnins.2020.611432

**Published:** 2020-12-21

**Authors:** Yajing Liu, Lushi Chen, Shuyun Huang, Chengguo Zhang, Zeping Lv, Jiali Luo, Pan Shang, Yukai Wang, Haiqun Xie

**Affiliations:** ^1^Department of Neurology, First People’s Hospital of Foshan, Foshan, China; ^2^National Research Center for Rehabilitation Technical Aids, Rehabilitation Hospital, Beijing, China

**Keywords:** Alzheimer’s disease, amnestic mild cognitive impairment, Pittsburgh Sleep Quality Index, sleep quality, plasma amyloid-β

## Abstract

**Study objectives:**

To investigate the extent to which sleep quality associated with plasma Aβ levels in amnestic mild cognitive impairment (aMCI) elderly.

**Methods:**

A total of 172 cognitively normal (NC) elderly and 133 aMCI elderly were included in this study. For the evaluation of sleep quality, the Pittsburgh Sleep Quality Index (PSQI) was used. Levels of plasma Aβ were determined by the sandwich enzyme-linked immunosorbent assay technique. Multivariable linear regression analysis was applied to evaluate associations between sleep quality and plasma Aβ levels after adjusting potential confounders.

**Results:**

Compared to NC subjects, participants with aMCI had a higher global PSQI score (8.72 ± 3.87 vs. 7.10 ± 3.07, *p* < 0.001). The global PSQI score was positively associated with plasma Aβ_42_ level in the aMCI group (β = 0.063, 95% CI 0.001–0.125, and *p* = 0.049) but not in the NC group (*p* > 0.05). Additionally, a higher global PSQI score was associated with a higher plasma Aβ_42_/Aβ_40_ ratio in both NC (β = 0.010, 95% CI 0.003–0.016, and *p* = 0.003) and aMCI groups (β = 0.012, 95% CI 0.005–0.018, and *p* < 0.001). The association between global PSQI score and plasma Aβ_42_/Aβ_40_ ratio was stronger in individuals with aMCI relative to the NC subjects (β = 0.076 vs. 0.030, *p* for interaction = 0.023).

**Conclusion:**

Poor sleep quality was associated with plasma Aβ_42_ and Aβ_42_/Aβ_40_ ratio, with a stronger effect among individuals with aMCI. A better understanding of the role of sleep in plasma Aβ levels in aMCI patients could lead to effective sleep-based intervention against the risk of Alzheimer’s disease.

## Introduction

Sleep disorders are commonly seen in older people and increase with age (Gadie et al., 2017). Growing evidences suggested that poor sleep quality tends to impair cognitive function (Karatsoreos et al., 2011; Yaffe et al., 2011; Kwon et al., 2015). A cohort study with 15,246 older people found that low sleep efficiency was associated with a high risk of memory impairment and poor cognitive function (Ma et al., 2019). Some case–control studies indicated that compared to cognitively healthy elderly, patients with mild cognitive impairment (MCI) had a higher prevalence of sleep disorder (Chiu et al., 2016; Palmer et al., 2018; Carnicelli et al., 2019).

Sleep disorder has been recognized as a significant risk factor for the development of Alzheimer’s disease (AD), which was attributed to amyloid-β (Aβ) deposition in the brain. Recent investigations verified that sleep/wake patterns might regulate Aβ peptide. Sleep decreased Aβ production and increased clearance, while wakefulness leads to an increase of Aβ production in the brain (Slats et al., 2013; Xie et al., 2013). These underlying associations were confirmed by several studies. Ooms et al. (2014) found that sleep deprivation enhanced cerebrospinal fluid (CSF) Aβ levels. Compared to sleep control, sleep deprivation increased overnight CSF Aβ levels by 25–30% via increased overnight Aβ production (Lucey et al., 2018). Furthermore, sleep deprivation promoted amyloid plaque formation in both humans and rodents (Kang et al., 2009; Ooms et al., 2014; Lucey et al., 2018; Shokri-Kojori et al., 2018). A prospective study with 70 cognitively healthy adults showed that poor sleep quality was associated with an increased Aβ burden in the brain (Spira et al., 2013).

The association between sleep quality and plasma Aβ has recently gained more interest. It has been confirmed that plasma Aβ levels correlated closely to the brain Aβ burden (Tzen et al., 2014; Nakamura et al., 2018). Aβ peptides involved dynamic interchange between the brain and periphery via transportation across the blood–brain barrier and blood–CSF barrier (Tarasoff-Conway et al., 2015). In light of these findings, few studies explored the association between sleep and plasma Aβ. Grimmer et al. (2020) reported that plasma Aβ levels decreased significantly after overnight sleep, and this reduction could be diminished by sleep disturbance in healthy adults. Wei et al. (2017) observed that after total sleep deprivation, plasma Aβ levels increased significantly in healthy young adults. A case–control study with 21 MCI patients suggested that disrupted slow-wave sleep was associated with plasma Aβ levels (Sanchez-Espinosa et al., 2014).

Despite these evidences, distinct sleep disorder patterns have not yet been well understood, especially in subjects with amnestic mild cognitive impairment (aMCI), and a subtype of MCI with a high risk of developing AD (Albert et al., 2011). Moreover, how sleep quality during a period of time influences plasma Aβ levels in aMCI individuals remains unclear. Understanding the effect of sleep disorder on aMCI may lead to effective sleep-based interventions for AD prevention. Given the acceptability and cost-effectiveness, blood-based plasma Aβ assay may be suitable for further investigating the causal roles of sleep disorder on Aβ levels among the large-scale general population. Hence, in this study, we aimed to primarily investigate the sleep characteristics of aMCI different from cognitively normal (NC) elderly and secondly evaluate the association between sleep quality and plasma Aβ in aMCI subjects.

## Materials and Methods

### Participants

In total, this study recruited 305 participants (aged 60–85), including 133 aMCI patients (mean age 69.68 ± 6.84) from Cognitive Disorders Clinics in the First People’s Hospital of Foshan and 172 NC subjects (mean age 68.59 ± 5.97) from community volunteers.

The diagnosis of aMCI (Petersen, 2004) was according to the following criteria: (1) subjective cognitive impairment and confirmed by an informant; (2) preserved activities of daily living confirmed by a clinician’s interviews; (3) multi-domain or a single-domain cognitive decline, and abnormal objective memory impairment determined by a cutoff of 1.5 standard deviations below education and age-matched specific norms by memory test; (4) global score of Clinical Dementia Rating (CDR) = 0.5 (Morris, 1993); and (5) absence of dementia according to the criteria of Diagnostic and Statistical Manual of Mental Disorders, Fourth Edition, and revised (DSM-IV-R). The inclusion criteria for NC participants were as follows: (1) cognitively normal confirmed by neuropsychological testing; and (2) CDR = 0.

The exclusion criteria included any of the following situations: (1) neurologic disorder, such as cerebral infarction or hemorrhage histories, Parkinson’s disease, epilepsy, or brain surgery; (2) autoimmune or peripheral vascular disease or cancer histories; (3) severe mood disorder (evaluated by the clinician according to the medical history and neuropsychological test); (4) obstructive sleep apnea; and (5) severe somatic disease, such as liver or kidney failure, and severe cardiopulmonary disease.

The ethics committee approved the research proposal in the First People’s Hospital of Foshan Research Ethics Board. We obtained written informed consent from the participants at enrollment.

### Plasma Aβ Assessment

Blood samples (7 ml) were collected in K3-Ethylenediaminetetraacetic acid (EDTA) tubes after overnight fasting. The blood was then centrifuged (3,000 rpm, 10 min, and 4°C), and the top layer containing plasma was moved into the Eppendorf tube. The plasma was stored at −80°C in 90 min after venipuncture. We used the sandwich enzyme-linked immunosorbent assay (ELISA) technique to assay the levels of plasma Aβ (Pesini et al., 2012). Aβ_40_ and Aβ_42_ in plasma were assayed using Human/Rat β Amyloid (40/42) ELISA Kit (Wako, Japan). Briefly, 100 μl of standards and plasma samples were added, which were then incubated with plate seal overnight at 4°C. One hundred microliters of the HRP-conjugated Antibody Solution was added, and the plate was sealed for 1 h at 4°C. Subsequently, 100 μl of TMB Solution was added, and then the plate was sealed, and incubated at room temperature in the dark. After 30 min, 100 μl of Stop Solution was added. The absorbance was read at 450 nm with a microplate reader. We used synthetic Aβ_40_ and Aβ_42_ peptides to establish standard curves, and we determined the concentration of Aβ.

### Sleep Assessment

The Pittsburgh Sleep Quality Index (PSQI) was used to assess sleep quality (Buysse et al., 1989). All of the participants completed the PSQI on the day of blood collection and confirmed that the quality of nighttime sleep prior to sampling was consistent with the description in the scale, with no significant fluctuation. PSQI is a recognized, self-administered questionnaire. It comprises 19 items that reflect subjective sleep quality, sleep latency, sleep duration, sleep efficiency, sleep disturbance, daytime dysfunction, and the use of sleeping medication. The scores of these seven components were reassigned, each weighted equally on the 0–3 scale. Scores of seven components were then summed to a yield a global PSQI score (0–21 scale), with higher scores indicating worse sleep quality.

### Medical and Cognitive Assessment

Data on demographic characteristics, medical history, lifestyle, physical measurements, and biochemical blood measurements were collected during health examinations. Participants completed a neuropsychological test battery, including Mini-Mental State Examination (MMSE) and the Chinese version of Montreal Cognitive Assessment (MoCA) to assess global cognition. Additionally, the Stroop’s Color Word Test (SCWT) was used to evaluate the executive function, and the Boston Naming Test was used to determine the language. Then, the Symbol Digit Modalities Test (SDMT) was used to assess attention; the Auditory Verbal Learning Test-Huashan version (AVLT-H) was used to evaluate memory. Furthermore, the geriatric depression scale (GDS) score was collected to adjust for the effects of subsyndromal depression.

### Statistical Analysis

The ratio of Aβ_42_/Aβ_40_ was log-transformed to correct skewness. The characteristics were compared between groups using Student’s *t* test for continuous variables and the χ^2^ test or Fisher’s exact test for the categorical variables. Multivariable linear regression analysis was used to estimate the effect values (β) and 95% confidence intervals (CIs) to examine the extent to which sleep quality associated with plasma Aβ variables. Three different models were tested: an unadjusted model, Model I, and Model II. Model I was adjusted for age and gender. Model II additionally included apolipoprotein E (APOE) ε4, education level, body mass index, exercise frequency, diabetes, hypertension, and the score of GDS. Sleep quality was stratified into tertile and then modeled as continuous variables to test for trends. Test for effect modification by the group employed interaction terms. Analyses were conducted by the statistical software packages R^1^ (The R Foundation) and Empower Stats^2^ (X&Y solutions, Inc., Boston, MA, United States). *p* value < 0.05 was considered statistically significant.

## Results

The descriptive characteristics of the study population is shown in Table 1. Compared to NC (aged 60–85), participants with aMCI (aged 61–85) had lower levels of education (*p* = 0.003) and lower total scores of MMSE (24.43 ± 2.37 vs. 26.91 ± 1.73, *p* < 0.001) and MoCA (19.75 ± 3.38 vs. 23.74 ± 2.89, *p* < 0.001). Additionally, plasma Aβ_42_ level (3.41 ± 1.30 vs. 3.06 ± 1.20, *p* = 0.013) and Aβ_42_/Aβ_40_ ratio (−1.06 ± 0.14 vs. −1.11 ± 0.12, *p* < 0.001) in participants with aMCI were higher than those in the NC group. Plasma Aβ_40_ level, the GDS score, body mass index, systolic blood pressure, diastolic blood pressure, fasting blood glucose, triglyceride, total cholesterol, high-density lipoprotein, low-density lipoprotein, APOEε4, hypertension, diabetes, and exercise frequency did not differ between NC and aMCI groups (*p* > 0.05).

**TABLE 1:** Characteristics of the study population.

	Overall (*N* = 305)	NC (*N* = 172)	aMCI (*N* = 133)	*p* value
Age (years)	69.07 ± 6.37	68.59 ± 5.97	69.68 ± 6.84	0.105
Male, *n* (%)	123 (40.33)	76 (44.19)	47 (35.34)	0.118
Aβ_40_ (pmol/L)	38.72 ± 13.40	38.92 ± 14.03	38.47 ± 12.59	0.772
Aβ_42_ (pmol/L)	3.21 ± 1.25	3.06 ± 1.20	3.41 ± 1.30	0.013
Aβ_42_/Aβ_40_*	−1.09 ± 0.14	−1.11 ± 0.12	−1.06 ± 0.14	<0.001
MMSE	25.83 ± 2.38	26.91 ± 1.73	24.43 ± 2.37	<0.001
MoCA	22.02 ± 3.68	23.74 ± 2.89	19.75 ± 3.38	<0.001
GDS	3.34 ± 2.05	3.17 ± 1.93	3.55 ± 2.19	0.115
BMI (kg/m^2^)	23.41 ± 2.98	23.66 ± 2.86	23.09 ± 3.10	0.105
SBP (mmHg)	133.55 ± 18.18	133.65 ± 18.43	133.42 ± 17.91	0.917
DBP (mmHg)	75.05 ± 11.19	75.84 ± 11.47	73.99 ± 10.76	0.165
FBS (mmol/L)	5.20 ± 1.20	5.27 ± 1.41	5.12 ± 0.83	0.296
TG (mmol/L)	1.49 ± 1.11	1.56 ± 1.35	1.39 ± 0.69	0.192
CH (mmol/L)	5.04 ± 0.97	5.00 ± 1.01	5.09 ± 0.93	0.413
HDL (mmol/L)	1.38 ± 0.35	1.36 ± 0.35	1.40 ± 0.36	0.273
LDL (mmol/L)	2.83 ± 0.80	2.80 ± 0.82	2.88 ± 0.79	0.397
APOE ε4, *n* (%)	43 (14.10)	21 (12.21)	22 (16.54)	0.281
HBP, *n* (%)	175 (57.95)	99 (57.56)	76 (58.46)	0.875
DM, *n* (%)	42 (13.77)	24 (13.95)	18 (13.53)	0.916
Exercise, *n* (%)^&^	220 (72.13)	126 (73.26)	94 (70.68)	0.618
Education level (years)				0.003
0, *n* (%)	10 (3.28)	2 (1.16)	8 (6.02)	
1–6, *n* (%)	90 (29.51)	42 (24.42)	48 (36.09)	
≥7, *n* (%)	205 (67.21)	128 (74.42)	77 (57.89)	

*Data expressed as mean ± standard deviation or percentage (%).*

^&^Exercise more than two times per week.

*Variables were log-transformed in the analyses.

*Aβ, amyloid-β; Aβ_42_/Aβ_40_, the ratio of Aβ_42_–Aβ_40_; MMSE, mini-mental state examination; MoCA, Montreal Cognitive Assessment; GDS, Geriatric Depression Scale; BMI, body mass index; SBP, systolic blood pressure; DBP, diastolic blood pressure; FBS, fasting blood glucose; TG, triglyceride; CH, total cholesterol; HDL, high-density lipoprotein; LDL, low-density lipoprotein; UA, uric acid; Cr, creatinine; APOE, apolipoprotein E; HBP, hypertension; DM, diabetes.*

Table 2 shows the sleep characteristics of the aMCI and NC subjects. Compared to NC subjects, aMCI patients had higher global PSQI scores (8.72 ± 3.87 vs. 7.10 ± 3.07, *p* < 0.001), which suggested worse sleep quality. It mainly reflected worse subjective sleep quality, long sleep latency, short sleep duration, low sleep efficiency, and more frequent sleep disturbances.

**TABLE 2:** Sleep quality of the study population.

Sleep variable	NC (*N* = 172)	aMCI (*N* = 133)	*p* value
Subjective sleep quality			0.003
Very good, *n* (%)	31 (18.13)	12 (9.09)	
Fairly good, *n* (%)	96 (55.56)	71 (53.03)	
Fairly bad, *n* (%)	44 (25.73)	41 (31.06)	
Very bad, *n* (%)	1 (0.58)	9 (6.82)	
Sleep latency			0.006
≤15 min, *n* (%)	69 (40.12)	39 (29.55)	
16–30 min, *n* (%)	76 (44.19)	52 (38.64)	
31–60 min, *n* (%)	21 (12.21)	28 (21.21)	
≥60 min, *n* (%)	6 (3.49)	14 (10.61)	
Sleep duration			0.001
>7 h, *n* (%)	37 (21.51)	17 (12.78)	
6–7 h, *n* (%)	80 (46.51)	53 (39.85)	
5–6 h, *n* (%)	51 (29.65)	47 (35.34)	
<5 h, *n* (%)	4 (2.33)	16 (12.03)	
Sleep efficiency	78.35 ± 10.82%	73.06 ± 12.08%	<0.001
>85%, *n* (%)	39 (22.67)	19 (14.39)	
75–84%, *n* (%)	68 (39.53)	38 (28.79)	
65–74%, *n* (%)	47 (27.33)	40 (29.55)	
<65%, *n* (%)	18 (10.47)	36 (27.27)	
Sleep disturbances			0.047
Not at all, *n* (%)	5 (2.91)	0 (0.00)	
At least once per week, *n* (%)	167 (97.09)	133 (100.00)	
Daytime dysfunction			0.994
Not at all, *n* (%)	80 (46.78)	59 (46.83)	
At least once per week, *n* (%)	92 (53.22)	67 (53.17)	
Sleeping medication			0.558
Not at all, *n* (%)	147 (85.47)	116 (87.72)	
Used, *n* (%)	25 (14.53)	17 (12.78)	
Global PSQI score	7.10 ± 3.07	8.72 ± 3.87	<0.001
T1 (1–5)^#^, *n* (%)	61 (35.47)	26 (19.55)	
T2 (6–8), *n* (%)	50 (29.07)	46 (34.59)	
T3 (9–19), *n* (%)	61 (35.47)	61 (45.86)	

*Pittsburgh Sleep Quality Index (PSQI) consisted of subjective sleep quality, sleep latency, sleep duration, sleep efficiency, sleep disturbance, daytime function, and use of sleeping medication. The scores of these seven components were reassigned, each weighted equally on 0–3 scale.*

*Data expressed as mean ± standard deviation or percentage (%).*

*^#^The low tertile of the global PSQI score, range of 1–5 scale.*

Table 3 shows the association between the global PSQI score and plasma Aβ. The global PSQI score was positively associated with plasma Aβ_42_ level in the aMCI group (β = 0.063, 95% CI 0.001–0.125, and *p* = 0.049) but not in the NC group (*p* > 0.05). After stratifying the global PSQI score into tertile, compared to those in tertile 1 (score < 5), participants in tertiles 2–3 had a higher plasma Aβ_42_ level in the aMCI group (tertile 2: β = 0.652, 95% CI 0.020–1.284, and *p* = 0.046; tertile 3: β = 1.093, 95% CI 0.461–1.724, and *p* < 0.001). We further tested the trends and found that the global PSQI score was positively associated with plasma Aβ_42_ level in the aMCI group (*p* < 0.001). Additionally, a high global PSQI score was associated with a high plasma Aβ_42_/Aβ_40_ ratio in both NC (β = 0.010, 95% CI 0.003–0.016, and *p* = 0.003) and aMCI groups (β = 0.012, 95% CI 0.005–0.018, and *p* < 0.001). Compared to those with low score (tertile 1), participants with a high score (tertiles 2–3) had higher plasma Aβ_42_/Aβ_40_ ratio in both NC (tertile 2: β = 0.046, 95% CI 0.000–0.093, and *p* = 0.046; tertile 3: β = 0.060, 95% CI 0.015–0.106, and *p* = 0.010) and aMCI groups (tertile 2: β = 0.109, 95% CI 0.042–0.176, and *p* = 0.002; tertile 3: β = 0.161, 95% CI 0.094–0.228, and *p* < 0.001). After adjusting for confounders and potential mediators, these associations also persisted. The further test of trend showed that global PSQI score was positively associated with plasma Aβ_42_/Aβ_40_ ratio in both NC (β = 0.030, 95% CI 0.008–0.053, and *p* = 0.010) and aMCI (β = 0.076, 95% CI 0.044–0.109, and *p* < 0.001) groups. Moreover, interaction terms were used to test effect modification by group classification (aMCI vs. NC). Patients with aMCI had a stronger association of global PSQI score (tertile) with plasma Aβ_42_ level and Aβ_42_/Aβ_40_ ratio relative to the NC subjects (β = 0.076 vs. 0.030, *p* for interaction = 0.023). There was no association between global PSQI score and plasma Aβ_40_ level in both NC and aMCI groups, even after adjusting for confounders and potential mediators (data not shown).

**TABLE 3:** Association between sleep quality and plasma Aβ levels.

Variable	Unadjusted model	Model I	Model II
			
	(Frame1)β (95% CI) *p* value	(Frame2)β (95% CI) *p* value	(Frame3)β (95% CI) *p* value
**Aβ_42_**			
**NC**			
Global PSQI score	0.021 (−0.037, 0.079) 0.484	0.025 (−0.035, 0.084) 0.419	0.029 (−0.031, 0.089) 0.348
T1 (1–5)^#^	Reference	Reference	Reference
T2 (6–8)	0.368 (−0.077, 0.814) 0.107	0.351 (−0.098, 0.800) 0.127	0.401 (−0.050, 0.853) 0.083
T3 (9–19)	0.146 (−0.277, 0.568) 0.499	0.157 (−0.272, 0.587) 0.473	0.173 (−0.266, 0.612) 0.441
*p* for trend	0.067 (−0.146, 0.280) 0.539	0.080 (−0.135, 0.296) 0.465	0.089 (−0.132, 0.309) 0.432
**aMCI**			
Global PSQI score	0.059 (0.003, 0.115) 0.042	0.061 (0.003, 0.118) 0.041	0.063 (0.001, 0.125) 0.049
T1 (1–5)	Reference	Reference	Reference
T2 (6–8)	0.658 (0.061, 1.255) 0.033	0.669 (0.066, 1.272) 0.031	0.652 (0.020, 1.284) 0.046
T3 (9–19)	1.055 (0.484, 1.626) < 0.001	1.063 (0.483, 1.644) < 0.001	1.093 (0.461, 1.724) < 0.001
*p* for trend	0.507 (0.232, 0.783) < 0.001	0.511 (0.230, 0.791) < 0.001	0.531 (0.224, 0.837) < 0.001
*p* for interaction by group^&^	0.013	0.013	0.009
Aβ_42_/Aβ_40_*			
**NC**			
Global PSQI score	0.009 (0.003, 0.015) 0.003	0.009 (0.003, 0.015) 0.003	0.010 (0.003, 0.016) 0.003
T1 (1–5)	Reference	Reference	Reference
T2 (6–8)	0.041 (−0.005, 0.087) 0.081	0.041 (−0.005, 0.087) 0.061	0.046 (0.000, 0.093) 0.046
T3 (9–19)	0.058 (0.014, 0.101) 0.010	0.059 (0.015, 0.103) 0.010	0.060 (0.015, 0.106) 0.010
*p* for trend	0.029 (0.007, 0.051) 0.010	0.029 (0.007, 0.051) 0.009	0.030 (0.008, 0.053) 0.010
**aMCI**			
Global PSQI score	0.009 (0.003, 0.015) 0.004	0.009 (0.003, 0.016) 0.004	0.012 (0.005, 0.018) < 0.001
T1 (1–5)	Reference	Reference	Reference
T2 (6–8)	0.099 (0.035, 0.164) 0.003	0.100 (0.035, 0.165) 0.003	0.109 (0.042, 0.176) 0.002
T3 (9–19)	0.138 (0.076, 0.200) < 0.001	0.141 (0.078, 0.203) < 0.001	0.161 (0.094, 0.228) < 0.001
*p* for trend	0.064 (0.034, 0.094) < 0.001	0.066 (0.035, 0.096) < 0.001	0.076 (0.044, 0.109) < 0.001
*p* for interaction by group^&^	0.055	0.049	0.023

^#^The low tertile of the global PSQI score, range of 1–5 scale.

*Variables were log-transformed in the analyses.

*^&^Interaction of group (aMCI vs. NC) on global PSQI score (tertile) and plasma Aβ.*

*Aβ_42_/Aβ_40_, the ratio of Aβ_42_–Aβ_40_; CI, confidence interval.*

*Model I: adjusted for age and gender.*

*Model II: Model I plus adjusted for APOEε4, education level, body mass index, exercise frequency, diabetes, hypertension, and geriatric depression scale score.*

## Discussion

The present study evaluated the extent to which sleep quality was associated with plasma Aβ levels in aMCI elderly. Poor sleep quality, as reflected by a high global PSQI score, was associated with increased plasma Aβ_42_ level in the aMCI group but not the NC group. A high Aβ_42_/Aβ_40_ ratio was associated with poor sleep quality, with a stronger effect among aMCI participants. Additionally, compared with normal control, aMCI subjects had worse sleep quality, indicated by worse subjective sleep quality, shorter sleep duration, longer sleep latency, lower sleep efficiency, and more sleep disturbances.

Growing evidence suggested that individuals with cognitive impairment had poor sleep quality relative to cognitively normal subjects. An interview survey with 2,413 elderlies in Taiwan reported that participants with cognitive impairment had a higher prevalence of self-reported sleep disturbances (Chiu et al., 2016). Palmer et al. (2018) showed that participants with MCI had a 3.2 higher odds of poor sleep measured by Sleep Continuity in Alzheimer’s disease Scale. Recent studies showed that MCI patients had a disrupted sleep with decreased rapid eye movement sleep and cyclic alternating pattern rate (Carnicelli et al., 2019), as well as poorer spindle and K-complex activities (Liu et al., 2020). Results in our study were consistent with previous studies. Importantly, we further characterized patterns of sleep quality and provided more detailed sleep disorder patterns, including subjective sleep quality, sleep duration, sleep latency, sleep efficiency, as well as the prevalence of sleep disturbances, which were not previously reported for individuals with aMCI.

In this study, we revealed that poor sleep quality was associated with increased plasma Aβ_42_ level in aMCI patients, as well as Aβ_42_/Aβ_40_ ratio in both NC and aMCI subjects. The underlying mechanism may be due to the following evidences. Firstly, the effect of sleep quality on Aβ mainly correlated with neuronal activity and synaptic strength. During wakefulness, neuronal activity increases and releases soluble Aβ. Conversely, during sleep, neuronal activity decreases, and Aβ production reduces (Cirrito et al., 2005). Thus, it is conceivable that sleep could decrease Aβ levels in the brain. Secondly, Aβ peptides were interchanged dynamically between the brain and periphery (Tarasoff-Conway et al., 2015). In light of these findings, plasma Aβ levels may be subject to sleep–wake states. Poor sleep quality, with an increased neuronal activity (Krueger et al., 2008; Vyazovskiy et al., 2009), might diminish physiological reduction of Aβ and lead to a relatively high level of plasma Aβ.

The association between sleep quality and plasma Aβ was different in NC and aMCI subjects. Our results for the first time to date suggested a high Aβ_42_/Aβ_40_ ratio, and increased Aβ_42_ levels were associated with poor sleep quality, with a stronger effect among aMCI participants relative to NC elderly. The underlying mechanism may be explained by recent findings. The relationship between poor sleep and Aβ accumulation is bidirectional (Ju et al., 2014). Poor sleep might increase Aβ levels in the brain. In particular, Aβ_42_ has long been recognized as a hydrophobic isoform with a tendency toward to form hard-to-clear aggregates (Tarasoff-Conway et al., 2015), which in turn increases the risk of amyloid plaque aggregation. Once amyloid plaques have formed, sleep–wake functions and circadian rhythms are disrupted (Roh et al., 2012; Ju et al., 2013). In comparison to healthy elderly, individuals with aMCI had a worse sleep quality in the present study. Additionally, previous evidence indicated that aMCI subjects had higher CSF Aβ_42_ levels, as well as more amyloid plaque formation than healthy elderly (Visser et al., 2009; Hanon et al., 2018; Knezevic et al., 2018). As a result, the bidirectional relationship between poor sleep and Aβ_42_ levels in the brain was more significant in individuals with aMCI. Given that Aβ peptides dynamically interchanged between the brain and periphery, the extent to which poor sleep quality associated with high plasma Aβ_42_ level and Aβ_42_/Aβ_40_ ratio in aMCI patients might be more significant than that in the NC subjects.

In this study, worse sleep quality was associated with a higher plasma Aβ_42_/Aβ_40_ ratio, but not Aβ_42_ level in the NC subjects. It is possible that the concentration of Aβ_42_ in plasma is much lower than that in CSF (Hanon et al., 2018). Evidence indicated that compared to the single peptide level in plasma, the ratio of Aβ_42_/Aβ_40_ had a higher sensitivity in predicting the Aβ burden in the brain (Nakamura et al., 2018).

This study has limitations worth noting. Firstly, sleep quality was evaluated with PSQI, a self-report, which may be less objective than polysomnography. Secondly, participants who had suspected obstructive sleep apnea syndrome were ruled out based on the medical history, as well as on their response to the question “cough or snore loudly” or “cannot breathe comfortably” during sleep in the PSQI. PSQI is a measurement of sleep quality; however, the sensitivity and accuracy in identifying sleep apnea syndrome are worse than polysomnography. It is necessary to apply more scientific wearable sleep monitoring equipment to determine the relationship between sleep and plasma Aβ in large-sized cohorts in the future. Thirdly, we did not detect CSF Aβ levels synchronously. Examining Aβ level in both CSF and plasma may be better to understand the effect of sleep duration and efficiency on Aβ metabolism, namely, how sleep disorder influences Aβ production, and how Aβ is cleared from the brain via the brain and periphery interchange pathway.

The present study provided evidence that poor sleep quality was associated with a high level of plasma Aβ in no-demented elderly, especially aMCI patients. These findings have significant implications for the prevention strategies of AD. As sleep quality can be intervened with drugs and physical activities, interventions to improve sleep quality may be conducive to delay the progression of aMCI to AD, altering the risk of AD onset. Additionally, in comparison to PET imaging and CSF detection, plasma Aβ assay is more cost-effective and less invasive, and thus might serve as an available tool for dynamic monitoring Aβ levels. Thereby, our study provided a significant basis for using plasma Aβ as a convenient tool to monitor and evaluate the effectiveness of sleep-based interventions. Moreover, PSQI has demonstrated consistent internal reliability and construct validity for sleep evaluation (Spira et al., 2012). Assessing sleep quality with PSQI is more convenient and cost-effective, reflecting usual sleep habits and quality without interference with the environment, thus allowing for assessing sleep quality in large-sized cohorts. Our findings might be helpful to identify sleep disorder as a modifiable risk and predictive factor, as well as facilitate proposing early intervention strategies for AD prevention. Future studies would be more optimal by applying wearable sleep monitoring equipment to further explore the relationship between sleep and plasma Aβ.

## Conclusion

These findings indicated that compared to NC subjects, aMCI patients had worse sleep quality. Furthermore, poor sleep quality was associated with plasma Aβ_42_ and Aβ_42_/Aβ_40_ ratio, with a stronger effect among individuals with aMCI. A better understanding of the sleep characteristics and the role of sleep in plasma Aβ levels in aMCI patients could lead to effective sleep-based intervention against the risk of AD. Our findings might be helpful to identify modifiable risk and predictive factors and facilitate proposing early intervention strategies for AD prevention.

## Data Availability Statement

The raw data supporting the conclusions of this article will be made available by the authors, without undue reservation.

## Ethics Statement

The studies involving human participants were reviewed and approved by First People’s Hospital of Foshan Research Ethics Board. The patients/participants provided their written informed consent to participate in this study.

## Author Contributions

YL and LC contributed to the literature search, data analysis, and the draft of the manuscript. SH, CZ, and ZL contributed to the acquisition of data. JL and PS undertook laboratory detection. HX and YW contributed to the study design, study supervision, and the revisions of the manuscript. All authors contributed to the article and approved the submitted version.

## Conflict of Interest

The authors declare that the research was conducted in the absence of any commercial or financial relationships that could be construed as a potential conflict of interest.

## Footnotes

1 http://www.R-project.org

2 www.empowerstats.com
